# External Validation of the Oakland Score for Predicting Safe Discharge in Patients Presenting With Lower Gastrointestinal Bleeding at the William Harvey Hospital in the United Kingdom

**DOI:** 10.7759/cureus.55497

**Published:** 2024-03-04

**Authors:** James Whiteway, Stephanie Yim, Natalie Leong, Ankur Shah

**Affiliations:** 1 Department of General Surgery, East Kent Hospitals University NHS Foundation Trust William Harvey Hospital, Ashford, GBR; 2 Department of General Surgery, East Cheshire NHS Trust Macclesfield District General Hospital, Macclesfield, GBR

**Keywords:** bsg guidelines, nice guidelines, current guidelines, external validation, scoring systems, per rectal bleed, hospital discharge, lower gastrointestinal bleeding, safe discharge, oakland score

## Abstract

Introduction

Lower gastrointestinal bleeds (LGIB) are defined by having a bleeding point in the gastrointestinal tract beyond the ligament of Treitz. The most common causes include diverticular bleeds, tumours, and colitis. There are no National Institute for Health and Care Excellence (NICE) guidelines regarding safe discharge of patients with LGIB. The aim of this study was to investigate the effectiveness and safety of the Oakland score, as suggested by the British Society of Gastroenterology (BSG) guidelines, in patients presenting with LGIB at William Harvey Hospital.

Methods

Patients with LGIB who presented to Accident & Emergency or inpatient referral from January to December 2023 were included in this retrospective study. Data was extracted from patients’ Sunrise documentation. The Oakland score for each patient was calculated. Those with a score of ≤8 were deemed safe for discharge; those with a higher score were deemed unsuitable. Patients’ admission, discharges, and adverse outcomes, such as representation, blood transfusion, or further intervention, were investigated. Patients with no adverse outcomes were deemed to have had a safe discharge. The area under the receiver-operating characteristic curve (AUROC) for the Oakland score and adverse outcome (and therefore safe discharge) were calculated.

Results

A total of 123 patients were included. These led to a total of 144 LGIB presentations to the hospital. Twenty-nine patients had an Oakland score of ≤8; 21 (72.4%) cases were initially discharged with four representations (19.0%) and eight (27.6%) were admitted although none of these suffered from any adverse outcomes. For those who scored ≤8, 25 (86.2%) were therefore deemed to have had a safe discharge. A total of 115 had a score >8; 43 (37.4%) were initially discharged, 72 (62.6%) admitted and 41 (35.7%) experienced at least one adverse outcome including 16 (13.9%) representations, 21 (18.3%) blood transfusions, three (2.6%) surgical interventions and one (0.9%) endoscopic haemostasis. Out of the 115 cases which scored >8, 74 (64.3%) were deemed to have had a safe discharge. The AUROC for safe discharge was 0.84.

Conclusion

The Oakland score seems to be a safe and reliable tool for identifying LGIB patients who could be safely discharged home without hospital intervention. However, further research is required to assess whether a score of >8 could be used as many patients with a higher score did not experience adverse outcomes.

## Introduction

Lower gastrointestinal bleeds (LGIB) are defined as any bleeding point within the GI tract that is distal to the ligament of Treitz at the duodenal-jejunal flexure. Any bleeding point that is proximal to this would be considered as an Upper GI bleed [1,2]. There are several causes leading to an LGIB, most notably diverticular disease, inflammatory (Crohn’s disease or Inflammatory bowel disease), vascular conditions (angiodysplasia), colitis, tumours and iatrogenic (non-steroidal anti-inflammatory drugs, surgery, endoscopy) [2-4]. Risk factors include ageing and the use of anticoagulation medication such as apixaban [2,4,5].

In the United Kingdom, there are currently no National Institute for Health and Care Excellence (NICE) guidelines surrounding the safe discharge of patients with LGIB. However, in 2019, guidelines were published in the British Society of Gastroenterology by Oakland et al. They suggested using a risk assessment tool such as the Oakland score to aid in the decision-making surrounding the safe discharge of these patients [6].

The aim of this study was to investigate the effectiveness and safety of the Oakland score in patients presenting with LGIB at the William Harvey Hospital in Kent, United Kingdom.

## Materials and methods

The inclusion criteria for this retrospective cohort study were any adult patient (≥18 years old) with LGIB. We did not include patients who presented with malaena or haematemesis, which would be more indicative of an upper gastrointestinal bleed. Patients with LGIB were identified using the Careflow app under the acute general surgery admissions which included patients presenting to Accident & Emergency (A&E) or from ward referrals and was updated on a daily basis between January and December 2023.

Data was extracted from the patient documentation on Sunrise including patient demographics, use of anticoagulants, comorbidities, blood results, vital signs, interventions and diagnosis. The data was then entered into the Oakland score calculator on the MDCalc website to generate a score [7]. Patients were then stratified according to their score. Those with a score of less than or equal to 8 were deemed safe for discharge; those with a higher were deemed unsuitable for discharge. We then investigated, regardless of the score, who was admitted to the hospital. The patients were followed over the course of the study to see whether any of them suffered from adverse outcomes. These were defined as representation in the hospital with further per rectal (PR) bleeding within 28 days, the need for blood transfusion, in-hospital death directly linked to the PR bleeding and the need for further intervention such as surgery, endoscopic haemostasis, or mesenteric embolisation. These patients were deemed not to have a safe discharge which was the primary outcome of the study.

Statistical analysis was performed on Microsoft Excel (Microsoft® Corp., Redmond, WA, USA). Patients with missing data from vital signs, blood results or outcomes were excluded from the statistical analysis of the Oakland score. The ability of the Oakland score to predict adverse outcomes and safe discharge in LGIB patients was analysed using the area under the receiver operating characteristic (AUROC) curves. As seen elsewhere, a cut-off point of ≥0.8 was used in order for the diagnostic test to be considered as meaningful [8].

## Results

**Figure 1:**
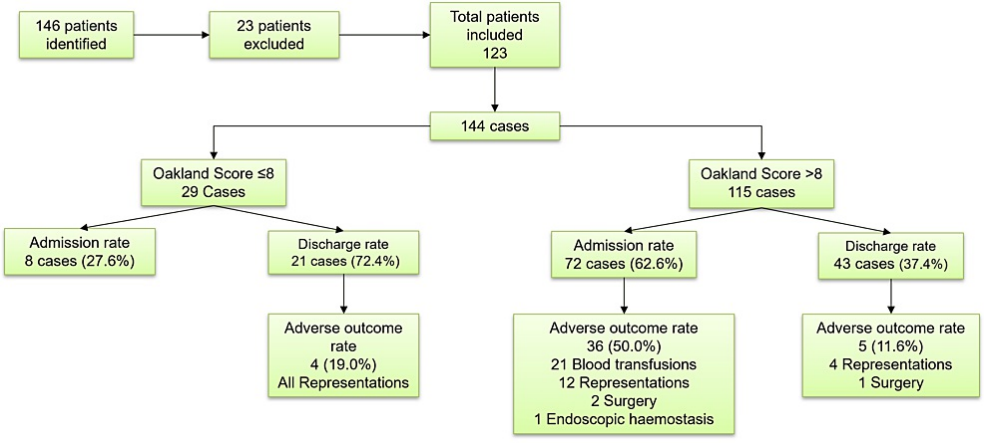
Flowchart of the patients included in the study stratified by the Oakland score

**Figure 2:**
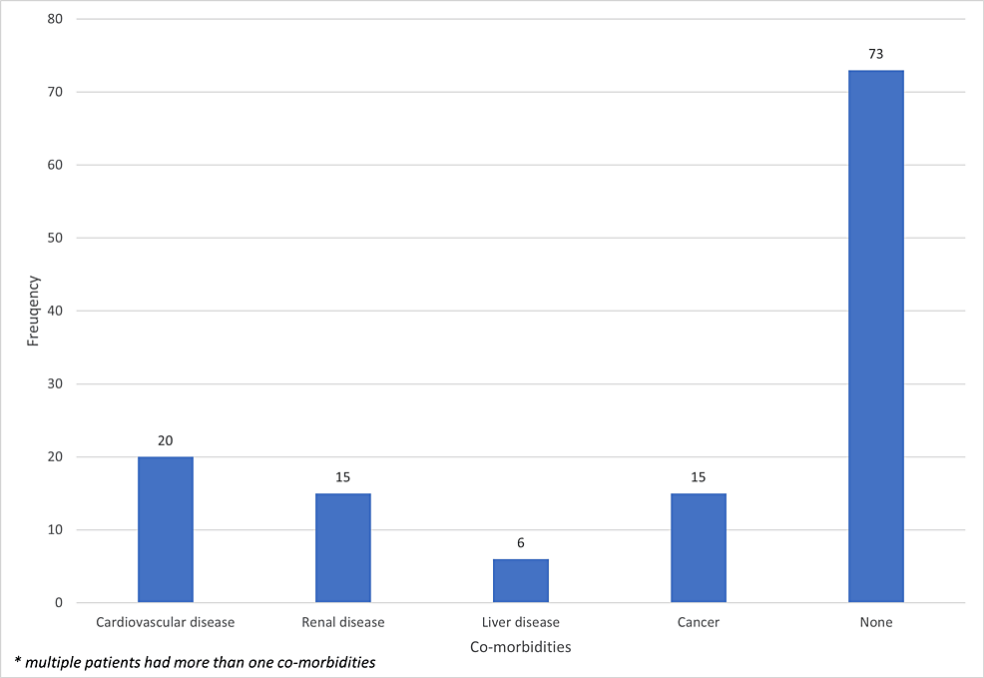
Graph of the comorbidities of the patients with lower gastrointestinal bleed

**Figure 3:**
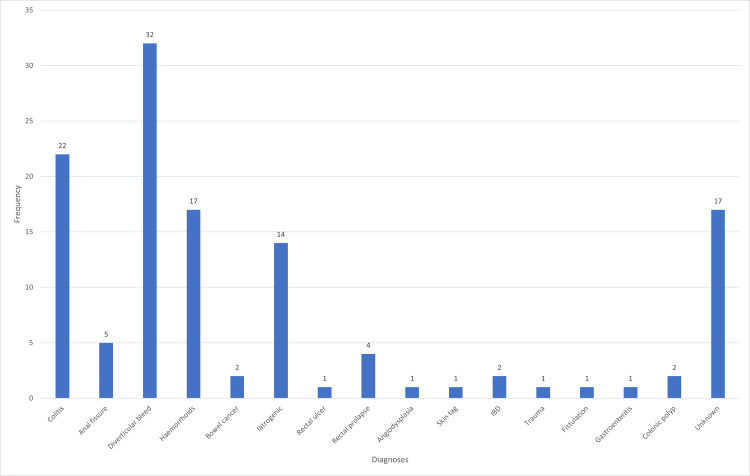
Breakdown of the diagnostic causes for lower gastrointestinal bleed

A total of 146 patients who accounted for 170 LGIB presentations to the hospital were identified. However, 23 patients had missing data regarding heart rate, blood pressure and/ or haemoglobin levels and were therefore excluded. In total, 123 patients were included in our study which amounted to 144 presentations to the hospital (see Figure 1) with 50 of the patients (40.7%) being male and 73 female (59.3%). The range of the patients’ ages was 19 to 98 years old with a median age was 68. Anticoagulants were prescribed in 24 patients (19.5%). The remaining 99 patients (80.5%) were not on anticoagulation (see Table 1). Regarding co-morbidities, 73 patients (59.3%) did not have any with the remaining 50 (40.7%) having at least one co-morbidity: 20 patients had cardiovascular disease (16.3%), 15 had renal disease (12.2%), six had liver disease (4.9%) and 15 had cancer (12.2%) (see Figure 2). The most common causes of PR bleed in these patients were mainly diverticular bleed with 32 cases (26.0%) followed by colitis (22, 17.9%), haemorrhoids (17, 13.8%) and iatrogenic (14, 11.4%). Importantly, 17 patients (13.8%) had an unknown cause of their PR bleed (see Figure 3 for full diagnosis breakdown).

**Table 1:** Patient characteristics

	n	%
Gender	123	
Male	50	40.7
Female	73	59.3
Median Age	68	
Oakland score		
0-8	26	21.1
9-35	97	78.9
Co-morbidities		
Cardiovascular disease	20	16.3
Renal disease	15	12.2
Liver disease	6	4.9
Cancer	15	12.2
None	73	59.3
Blood transfusion		
No	102	82.9
Yes	21	17.1
Anticoagulant use		
Yes	24	19.5
No	99	80.5
Further intervention		
Surgery	3	2.4
Mesenteric embolization	0	0.0
Endoscopic haemostasis	1	0.8
None	119	96.7

**Figure 4:**
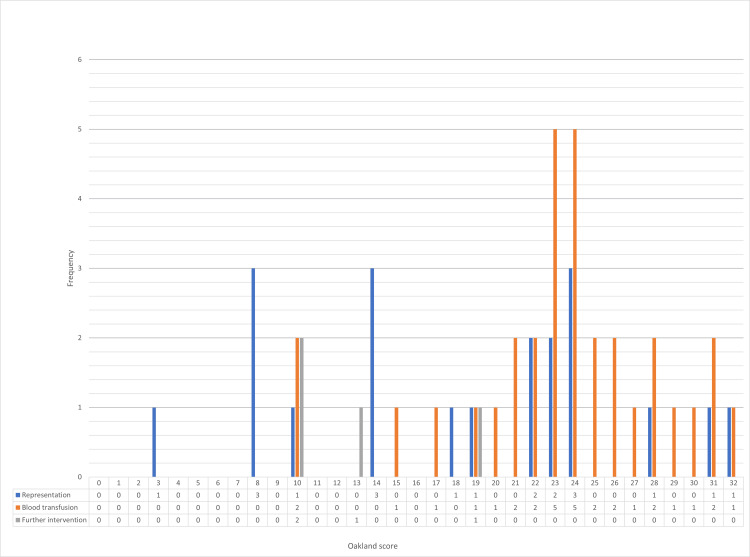
Bar graph showing the Oakland score and adverse outcomes

Twenty-nine cases (20.1%) had an Oakland score of less than or equal to 8, ranging from 1 to 8, while the remaining 115 cases (79.1%) had an Oakland score above 8, ranging from 9 to 32. Of those who had a score less than or equal to 8, eight cases were admitted (27.6%) and 21 (72.4%) led initially to a discharge with four cases (19.0%) representing back to the hospital. None of the admissions led to adverse outcomes. For those who scored ≤8, 25 (86.2%) were deemed to have had a safe discharge. Out of the 115 cases with a score higher than 8, 72 (62.6%) had an admission and 43 (37.4%) were initially discharged. There were 41 cases (35.7%) with at least one adverse outcome (see Figure 4). The latter included: 16 (13.9%) representations, 21 (18.3%) blood transfusions, three (2.6%) surgical interventions and one (0.9%) endoscopic haemostasis. No patient underwent mesenteric embolisation. Furthermore, not a single inpatient died directly because of PR bleeding. It is important to note however that seven of the patients did die of other causes over the course of the study. Out of the 115 cases which scored >8, 74 (64.3%) were deemed to have had a safe discharge.

**Figure 5:**
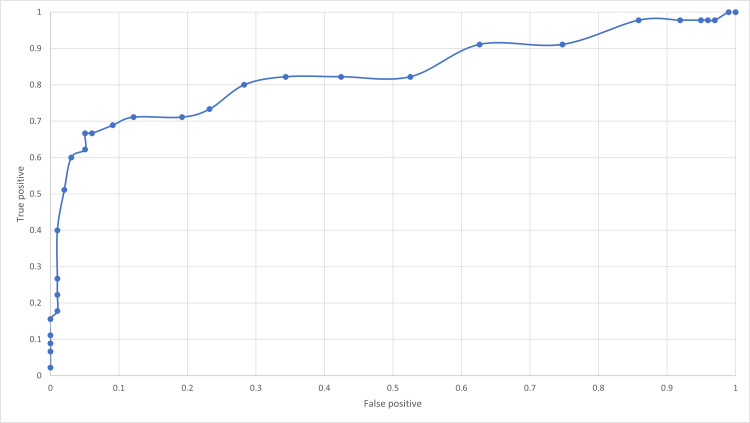
Receiver operating characteristic curve of the Oakland score for safe discharge

In our cohort, the AUROC for the ability of the Oakland score to predict safe discharge was 0.84 which suggests that this scoring system is a good predictor for safe discharge (Figure 5).

## Discussion

The study has shown that patients who were deemed to have a lower Oakland score (less than 8) were less likely to be admitted compared to those with a higher score (more than 8). It is important to note, however, that in the former group, eight of the patients were admitted to the hospital yet their score would have been deemed safe for them to be discharged. Seven out of the eight cases had Oakland scores on the higher end (7-8) with one patient only scoring 1. All of these patients were admitted for observation only and did not suffer any further rebleeds nor did they represent to the hospital even after discharge. Indeed, no further intervention was required such as blood transfusion or having to take the patient to the theatre to stop the bleeding. All patients who were admitted had self-resolving PR bleeds. Many of these patients (4) were suffering from diverticular bleeds followed by colitis (3) and one was still undergoing investigations at the time of writing. Only three patients suffered from comorbidities (all cardiovascular disease) with one of these also prescribed anticoagulants. When looking at the length of stay, the range was from less than 24 hours to six days. Indeed, the patient with the lowest score of 1 only stayed for one day.

For the remaining patients who were sent home immediately, four patients experienced adverse outcomes. They all represented to the hospital between 0-4 days, two of the patients represented on the same day. One who originally presented with PR bleed was also complaining of abdominal pain. According to the patient, this was more concerning to them than the actual PR bleed. CTAP revealed colitis. The other patient represented with further bleeding. CTAP showed diverticulitis and he was later discharged that day. The other two patients represented several days afterwards. One presented originally with a PR bleed secondary to haemorrhoids. Upon representation, CTAP was performed and showed diverticulitis. Their score was in fact lower compared to the previous presentation (at 6 from 8) and they were discharged with safety netting. The final patient represented four days later due to thrombosed haemorrhoids. Similarly, she represented with a lower score of 6 from 8. Given the pain she was in rectally due to her known diagnosis of haemorrhoids, she was taken to the emergency theatre list for examination under anaesthetic as a day case which revealed the above diagnosis and was later discharged that day. All of the patients that had represented with a score of less than or equal to 8 had stable observations. Only one patient suffered from a comorbidity (cardiovascular disease). The same patient was the only one as well to have anticoagulants prescribed. The majority of the patients with a minor bleed (17) had otherwise safe discharges. It could be argued that most of the patients with a low score had no complications and even those who were admitted only required observation and no further intervention.

On the other hand, with regards to the patients with life-threatening PR bleeds, 72 cases with an Oakland score of more than 8 were admitted to the hospital. Interestingly, 43 cases who scored more than 8 on the Oakland score did not lead to admission. The Oakland score ranged between 9 and 18 which could be seen as the midrange of scores. Of these patients, there were five cases with adverse outcomes. These included four representations to the hospital as a result of two diverticular bleeds, one rectal prolapse and one IBD, and one patient with fistulation requiring surgery. The remaining 38 patients were deemed safe for discharge. Many of these patients were suffering from bleeding haemorrhoids or anal fissures and are therefore subjected to the clinical judgement of the doctor. These causes could be considered less significant compared to diverticular disease. On the other hand, many of the patients who had a score larger than 8 who were admitted to the hospital suffered at least one or more adverse outcomes (36). Interestingly, it seems that the use of anticoagulants does not increase the chances of developing an adverse outcome with only seven out of 24 patients on anticoagulants suffering from adverse outcomes. Similarly, patients who suffered from comorbidities were not at an increased risk of developing adverse outcomes - only 16 patients out of 51 had co-morbidities and adverse outcomes. However, compared to other studies validating the Oakland score [9,10], the sample size of the cohort was smaller and therefore the relationship between anticoagulation use, comorbidities, and the risk of adverse outcomes could not be studied accurately.

As seen in other external validation studies such as the Oakland et al. or Fong study, the AUROC for the ability of the Oakland score to safe discharge in LGIB patients was 0.84 compared to 0.87 and 0.88, respectively [9,10]. This is therefore suggesting that the Oakland score is a good tool to predict safe discharge in this cohort of patients as the AUROC > 0.8 [8]. It was argued in the original study that the new Oakland score was superior to many of the other scoring systems including Rockall, Blatchford, STRATE, BLEED, NOBLADS and AIMS65 [11]. Indeed, a study by Tapaskar et al. compared these risk scores and found that the Oakland score was the best predictor for the severity of bleeding [12]. In 2022, there was a meta-analysis that compared these risk-stratifying scores. They looked at various outcomes including safe discharge, major bleeding, need for transfusion and need for haemostasis. The authors also tried to investigate mortality as an outcome, but there was insufficient data on the subject. The Oakland score was found to be the best predictor for safe discharge, major bleeding and need for transfusion [13]. As a predictor for mortality, a recent study by Yeon et al. showed that the AIMS65 score and ABC score were better at predicting 30-day mortality than the Oakland score [14]. It has been suggested in other studies that a higher cut of 10 or more could be used [9,10]. Indeed, 38 patients with a score >8 were safely discharged. A 2020 large prognostic study in the US identified a greater proportion of lower-risk patients while maintaining a sensitivity of 96% when they included a cut-off of 10 instead of 8. However, there were more adverse events [9]. Furthermore, the Fong study found that a threshold of ≤11 points resulted in a sensitivity of safe discharge at 97% [10]. Similar results were also seen in the 2021 Martin et al. study. They proposed that patients with an Oakland score of more than 8 with a systolic blood pressure (SBP) > 100 mmHg, HR < 100, and Hb > 13g/dL could be deemed safe to go home [15].

This study has several limitations. Firstly, in comparison to more severe LGIB, there were not as many patients within the Oakland score of ≤8 group - only 29 cases. Many of the patients that could have possibly fallen into this group were excluded from the study. This was because, at the East Kent University Hospital Trust, LGIB patients may be seen via the Surgical Emergency Assessment Unit (SEAU) pathway where they will bypass A&E. Unfortunately, this meant that many patients had missing observation data as these would usually not be uploaded onto Sunrise. As a result, it was not possible to calculate the Oakland score. Furthermore, patients have to be clinically stable in order to be accepted by the SEAU pathway; otherwise, they would have to be seen via A&E (which routinely uploads the observations to Sunrise). Secondly, patients were identified retrospectively through the general surgery acute admissions Careflow app. The list of patients changes daily to include all the patients that were either seen in A&E or inpatient referral for that day. The general surgery on-call team will usually save the list as a portable document format (PDF) in order for it to be accessed at a later date. However, this does not always happen and therefore it is possible that more patients with LGIB could have been missed. Finally, because of the above limitations, the study has a smaller sample size compared to other external validation studies although similar results were seen [9,10].

## Conclusions

The Oakland score seems to be a reliable tool for predicting safe discharge in low-risk LGIB patients. Currently, the cut-off point for patients to be safely discharged with an urgent outpatient intervention is an Oakland score of 8 or less. However, many of the higher-risk patients in this retrospective cohort study did not suffer any adverse events even when their score was larger than 8. Further research is therefore required to assess whether a higher cut-off point would be more appropriate such as a score of 10.

## References

[1] Ramaswamy RS, Choi HW, Mouser HC, Narsinh KH, McCammack KC, Treesit T, Kinney TB (2014). Role of interventional radiology in the management of acute gastrointestinal bleeding. World J Radiol.

[2] Strate LL (2005). Lower GI bleeding: epidemiology and diagnosis. Gastroenterol Clin North Am.

[3] Murphy B, Winter DC, Kavanagh DO (2019). Small bowel gastrointestinal bleeding diagnosis and management-A narrative review. Front Surg.

[4] Gunjan D, Sharma V, Rana SS, Bhasin DK (2014). Small bowel bleeding: a comprehensive review. Gastroenterol Rep (Oxf).

[5] Holster IL, Valkhoff VE, Kuipers EJ, Tjwa ET (2013). New oral anticoagulants increase risk for gastrointestinal bleeding: a systematic review and meta-analysis. Gastroenterology.

[6] Oakland K, Chadwick G, East JE (2019). Diagnosis and management of acute lower gastrointestinal bleeding: guidelines from the British Society of Gastroenterology. Gut.

[7] (2024). MDCalc. Oakland score for safe discharge after lower GI bleed. https://www.mdcalc.com/calc/10042/oakland-score-safe-discharge-lower-gi-bleed.

[8] Nahm FS (2022). Receiver operating characteristic curve: overview and practical use for clinicians. Korean J Anesthesiol.

[9] Oakland K, Kothiwale S, Forehand T (2020). External validation of the Oakland score to assess safe hospital discharge among adult patients with acute lower gastrointestinal bleeding in the US. JAMA Netw Open.

[10] Fong HY (2023). External validation of the Oakland score to assess safe hospital discharge among adult patients with acute lower gastrointestinal bleeding in an accident and emergency department in Hong Kong. Hong Kong J Emerg Med.

[11] Oakland K, Jairath V, Uberoi R (2017). Derivation and validation of a novel risk score for safe discharge after acute lower gastrointestinal bleeding: a modelling study. Lancet Gastroenterol Hepatol.

[12] Tapaskar N, Jones B, Mei S, Sengupta N (2019). Comparison of clinical prediction tools and identification of risk factors for adverse outcomes in acute lower GI bleeding. Gastrointest Endosc.

[13] Almaghrabi M, Gandhi M, Guizzetti L (2022). Comparison of risk scores for lower gastrointestinal bleeding: a systematic review and meta-analysis. JAMA Netw Open.

[14] Yeon SH, Moon HS, Choi SW, Kang SH, Sung JK, Jeong HY (2023). A comparative study of scoring systems that accurately predict the prognosis of lower gastrointestinal bleeding. Int J Colorectal Dis.

[15] Martin TA, Tewani S, Clarke L (2021). Factors associated with emergency department discharge, outcomes and follow-up rates of stable patients with lower gastrointestinal bleeding. Gastroenterol Res.

